# A Workflow Combining Machine Learning with Molecular Simulations Uncovers Potential Dual-Target Inhibitors against BTK and JAK3

**DOI:** 10.3390/molecules28207140

**Published:** 2023-10-17

**Authors:** Lu Liu, Risong Na, Lianjuan Yang, Jixiang Liu, Yingjia Tan, Xi Zhao, Xuri Huang, Xuecheng Chen

**Affiliations:** 1 Institute of Theoretical Chemistry, Jilin University, Changchun 130061, China; l_liu18@mails.jlu.edu.cn (L.L.); jixiang20@mails.jlu.edu.cn (J.L.); tanyj20@mails.jlu.edu.cn (Y.T.); 2 Collaborative Innovation Center of Henan Grain Crops, National Key Laboratory of Wheat and Maize Crop Science, College of Plant Protection, Henan Agricultural University, Zhengzhou 450002, China; nrs@henau.edu.cn; 3 Department of Medical Mycology, Shanghai Skin Disease Hospital, Tongji University School of Medicine, Shanghai 200443, China; lianjuanyang@163.com; 4 Department of Nanomaterials Physicochemistry, Faculty of Chemical Technology and Engineering, West Pomeranian University of Technology, Szczecin Piastów Ave. 42, 71-065 Szczecin, Poland; xchen@zut.edu.pl

**Keywords:** BTK, JAK3, machine learning, SHAP, virtual screening, molecular dynamics simulation

## Abstract

The drug development process suffers from low success rates and requires expensive and time-consuming procedures. The traditional one drug–one target paradigm is often inadequate to treat multifactorial diseases. Multitarget drugs may potentially address problems such as adverse reactions to drugs. With the aim to discover a multitarget potential inhibitor for B-cell lymphoma treatment, herein, we developed a general pipeline combining machine learning, the interpretable model SHapley Additive exPlanation (SHAP), and molecular dynamics simulations to predict active compounds and fragments. Bruton’s tyrosine kinase (BTK) and Janus kinase 3 (JAK3) are popular synergistic targets for B-cell lymphoma. We used this pipeline approach to identify prospective potential dual inhibitors from a natural product database and screened three candidate inhibitors with acceptable drug absorption, distribution, metabolism, excretion, and toxicity (ADMET) properties. Ultimately, the compound CNP0266747 with specialized binding conformations that exhibited potential binding free energy against BTK and JAK3 was selected as the optimum choice. Furthermore, we also identified key residues and fingerprint features of this dual-target inhibitor of BTK and JAK3.

## 1. Introduction

**Figure A1:**
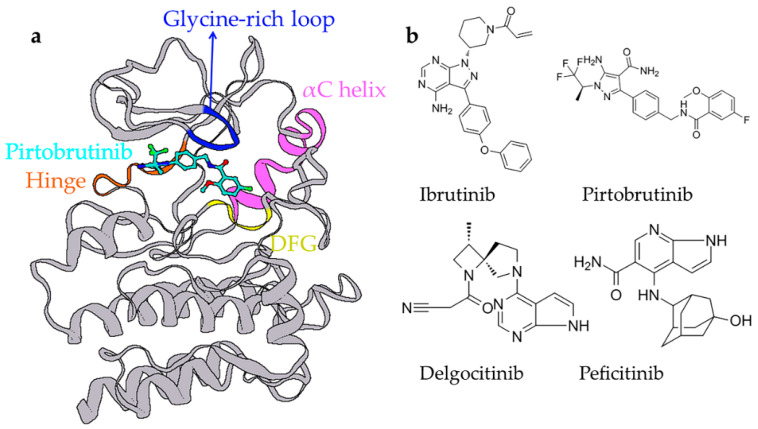
(**a**) The X-ray crystal structures of BTK and pirtobrutinib are shown as gray cartoon and cyan sticks, respectively. The key regions are marked with different colors. (**b**) Structures of several reported BTK and JAK3 inhibitors.

In the 20th century, the doctrine “one molecule, one target, one disease” served as a guiding principle for the pharmaceutical industry. However, this paradigm was recognized to be unsatisfactory for therapeutic effects of multifactorial diseases such as tumors and immune system diseases [1,2]. Therefore, it is crucial to discover drugs that simultaneously manipulate multiple targets and interrupt the pathogenesis process of multifactorial diseases [3]. Studies have highlighted the overexpression of kinases in many cancers [4], and different kinase inhibitors have gained popularity as potential antitumor agents [5]. However, concerns of drug resistance and off-targeting toxicity are yet unaddressed [6,7], and multitarget drugs that can overcome these limitations are warranted. For instance, Bruton’s tyrosine kinase (BTK) and Janus kinase 3 (JAK3) are two validated and therapeutically amenable targets to effectively treat B-cell lymphomas and can be used to develop a dual-target inhibitor [8]. As with most kinases, BTK and JAK3 share similar structures in the binding pocket, including the hinge (connecting C-terminal and N-terminal), glycine-rich loop (GRL), αC helix, and highly conserved DEG motif (Figure A1a) [9,10].

BTK belongs to the nonreceptor Tec tyrosine kinase family, widely expressed in hematopoietic cells. BTK plays an extremely important role in signaling through the Fcγ receptor (FcγR) and B-cell antigen receptor (BCR) [11,12], and its deregulation has been associated with many B cell-related malignancies such as multiple myeloma (MM) and lymphocytic leukemia (CLL) [13,14]. A few BTK inhibitors, including ibrutinib [15,16], orelabrutinib [17], and pirtobrutinib [18,19], have been approved by the US Food and Drug Administration (FDA), and several new ones are at different stages of trials (Figure A1b). Pirtobrutinib is a third-generation noncovalent inhibitor with better safety and improved selectivity for many B cell-derived diseases [18]. JAKs are members of the nonreceptor tyrosine kinase family that mediate growth factor production and cytokine and play crucial roles in immune signaling [20]. JAKs comprise tyrosine kinase TYK2, JAK1, JAK2, and JAK3. JAK3 exhibits a binding pocket region that is highly conserved with other JAK family kinases except at residues CYS909 and ALA966 [21,22]. JAK3 is mainly expressed in hematopoietic cells, and was proved to play a crucial role in the mediation of the antiapoptotic phosphoinositide 3-kinase (PI3K)-protein kinase B (AKT) pathway and survival of leukemic B-cell precursors [23,24,25]. Therefore, JAK3 is an appealing target for lymphoid malignancies and a potential target for autoimmune diseases, given its important functions in the immune system. The FDA has approved several drugs such as tofacitinib [26,27] and peficitinib [28] (Figure A1b). Given their synergistic effects, the simultaneous inhibition of the BTK/JAK3 signaling pathways can be an optimal therapy as compared with drugs against single targets [29,30]. A common issue with kinase inhibitors is toxic side effects. Due to their low toxicity and wide availability, natural products are a meaningful source for the exploration of BTK and JAK3 dual-target inhibitors. Since natural product inhibitors against BTK and JAK3 have been largely unreported, the research on active natural product inhibitors is promising and valuable. However, the restrictions of traditional screening methods have made it hard to keep up with the rapid pace of drug development.

Computer-aided drug design (CADD) has evolved into a necessary tool for drug discovery [31] and has remarkable potential for single-target discovery [32,33]. However, the selection of target combinations to achieve the desired efficacy is still challenging. Many computational derivative tools developed over the last few decades have successfully transformed molecular structural information from experimental data into a molecular characterization. The application of these algorithms to the drug discovery pipeline may reduce resource expenditures and provide a direction for the design of drugs specific for dual targets. Examples of BTK and JAK3 dual targets have been previously described where simultaneous inhibition of BTK and JAK3 not only effectively inhibited the signaling pathway of malignancy growth but also addressed the concern of drug resistance [30].

**Figure 1:**
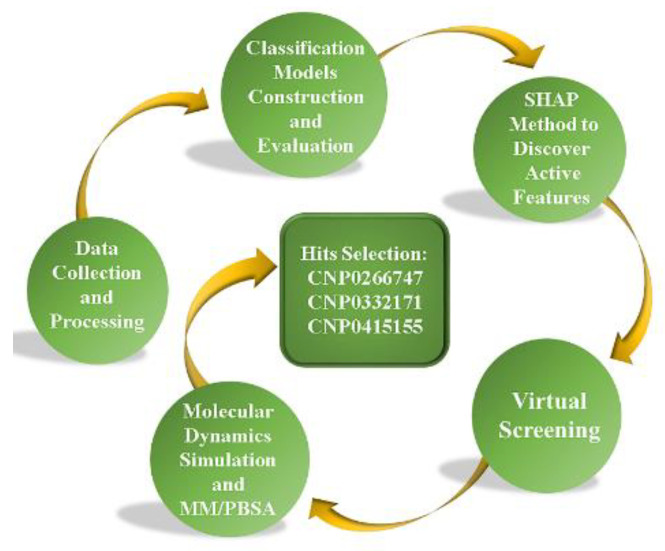
The pipeline proposed in this work for virtual screening of potential BTK and JAK3 dual inhibitors.

Herein, we introduce a general pipeline that integrates machine learning methods, the interpretable model SHapley Additive exPlanation (SHAP) [34], and molecular dynamics simulations for discovering potential dual inhibitors (Figure 1). We applied this pipeline to the discovery of potential dual inhibitors of BTK and JAK3 from a natural product database (the Coconut database is a generalized natural product database which has been consolidated from 53 different databases and the literature). The machine learning models were built using random forests (RFs), extra trees (ETs), and extreme gradient boosting (XGB) and validated for prediction of inhibitor activities. Later, we applied these three models to predict active compounds from the natural products to discover potential dual inhibitors of BTK and JAK3. We used the interpretable model SHAP to interpret the effect of individual active molecular fingerprint features on outcome prediction. Next, three compounds against BTK and JAK3 were identified via molecular dynamics and binding free energy. These compounds have potential to serve as dual-target inhibitors against BTK and JAK3. Finally, we selected the optimal compound CNP0266747 among the screened compounds as the most promising potential inhibitor.

## 2. Results

### 2.1. Spatial Diversity

**Figure 2:**
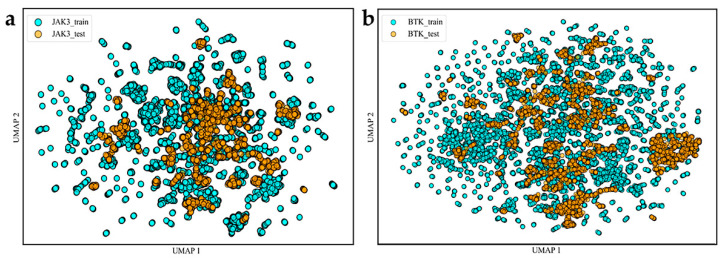
Diversity distribution of the modeling data. (**a**,**b**) Two-dimensional spatial distribution maps of BTK and JAK3, respectively.

The chemical spatial distributions reflect the reasonability of the data, which influences the model construction process. We evaluated the rationality of the data by calculating 2048 extended connectivity fingerprints (ECFPs) for the training and test sets. We applied the downscaling method of uniform manifold approximation and projection (UMAP) to visually represent their chemical spatial distribution [35]. As displayed in Figure 2, the UMAP diagram clearly demonstrates the wide chemical spatial distribution of the training and test data. The quality of the data is an important issue to consider before constructing machine learning models. Therefore, the high diversity in the training and test sets proves that our data have excellent robustness. Our analysis indicates that the compounds used to build this model were reasonable and differed in their chemical structures.

### 2.2. Establishment and Validation of Models

**Figure 3:**
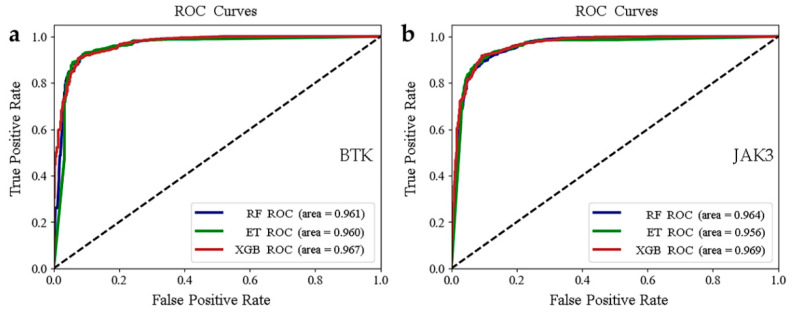
(**a**,**b**) Plots of ROC curves for the BTK and JAK3 test sets.

To obtain the optimal combination of super parameters, we used the Bayesian optimization method. We applied them to the classification models, employed three machine learning models (RF, ET, and XGB) for model construction, and performed tenfold cross-validation. The scores for each model are displayed in Table 1 and Table 2. The three classification models had high values (more than 0.9) of recall, precision, F1 score, and accuracy for the test set. In addition, we also plotted the receiver operating characteristic (ROC) curves of the three models for two targets and provided the corresponding area under the curve (AUC) values (Figure 3). The ROC curve is a significant indicator for evaluation of the prediction and classification capability of the models. The closer to 1 the AUC value is, the better its capacity for classification. As seen in Figure 3, the AUC values of the test set for RFs, ETs, and XGB were more than 0.95. Thus, the evaluation indicators of the three models were on the same level. Together, these results indicate that all of our models exhibit high predictive power and robust classification capabilities for the compounds. Therefore, we could apply these models to predict active molecules from the database.

### 2.3. Explanatory Analysis of Models

**Figure 4:**
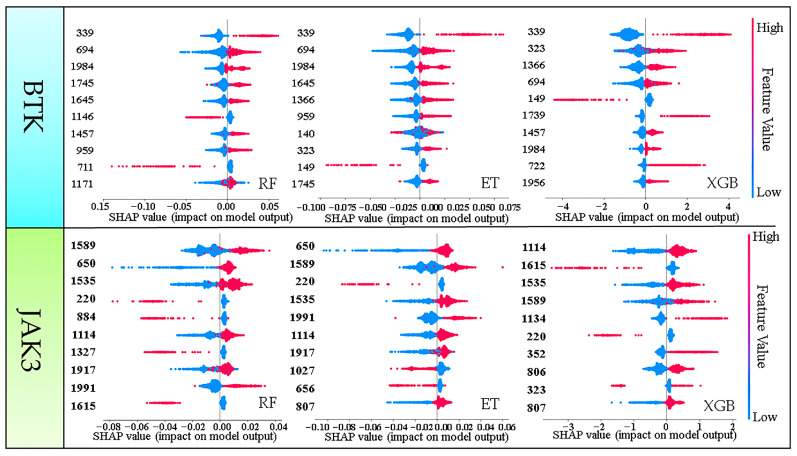
The scatter plot of feature density of the top 10 molecular fingerprint fragments. Red color means a sample point has a large Shapley value and blue color means a sample point has a small Shapley value.

As fingerprint space was used to construct machine learning models, it is possible to determine the biological activity of compounds from different chemical groups or molecular skeletons. To gain a better understanding of the importance of fingerprint fragments on the specific direction of decisions of the models, we employed a feature density scatter plot in SHAP as a holistic approach to interpretation. This scatter plot sorted the Shapley value of each feature into the corresponding position coordinates. As shown in Figure 4, the y-axis indicates the importance of the model’s predictive features and the x-axis shows the effect on model predictions (red color indicates the sample point has a large Shapley value and blue color indicates a small Shapley value). We combined the Shapley values with sample point colors to investigate the relationship between feature variation and decision direction. Shapley values greater than 0 indicate a positive impact, and vice versa for negative impact. Based on this description, we found fingerprint fragments with large Shapley values using the scatter plot (Figure 4).

BTK: Figure 4 shows the top 10 Shapley values of the fingerprint fragments. Only three fingerprint fragments, 339, 694, and 1984, appeared in the top 10 Shapley values of all three models, especially fingerprint fragment 339 (Table 3). A larger Shapley value increases the probability of the molecule being active. Therefore, the Shapley value of fingerprint fragment 339 was located at the top of the three models, which gathered our attention.

JAK3: The top 10 Shapley values of the fingerprint fragments are shown in Figure 4. Only the fingerprint fragments 1589, 1535, and 1114 appeared in the top 10 Shapley values of all three models (Table 3) and were the focus objects, especially 1589, which had the highest value of the three fingerprints. These findings not only provide an insight into the impact of fingerprint features on the model but also serve as the foundation for fragment-based drug design.

### 2.4. Virtual Screening

Given the same level of the predictive categorization capacity of the three machine learning models, we employed the equal weights of the RF, ET, and XGB models to screen the natural compounds from the Coconut database. To further increase the accuracy of classification and exclude redundant and incompatible molecules, we calculated fingerprints for the molecules from the database and eliminated those with correlation coefficients < 0.1. Finally, we obtained a dataset containing 123,398 molecules. The three models were used to virtually screen Coconut, which yielded 14,465 candidate inhibitors for further molecular docking experiments.

**Figure A2:**
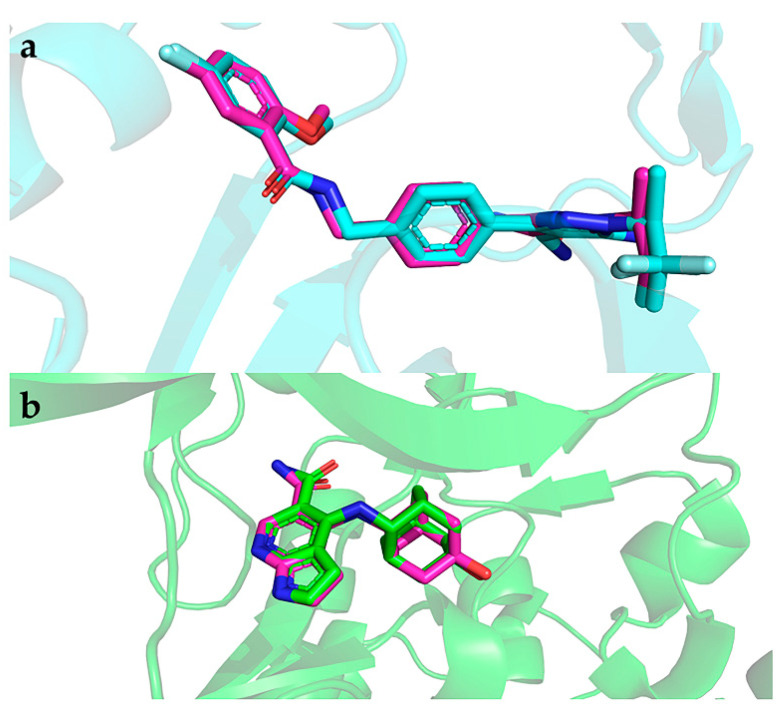
The superimposition of the redocked and co-crystallized ligands. (**a**) Cyan color indicates the redock compound and violet color indicates the co-crystallized compound of BTK. (**b**) Green color indicates the redocked compound and violet color indicates the co-crystallized compound of JAK3.

Molecular docking describes the pose and location of the ligand at the binding pocket of the protein. To validate the accuracy of the docking results, AutoDock Vina was used to redock the co-crystallized compounds of BTK (PDB ID: 8FLL) and JAK3 (PDB ID: 6AAK). In Figure A2, the two co-crystallized proteins reproduced the original docking’s consistent spatial orientation with affinities of −11.2 and −10.5 kcal/mol for BTK and JAK3, respectively. Thus, our docking results are reliable and can be applied to determine potential inhibitors of BTK and JAK3.

Next, we used AutoDock Vina to complete the docking operations of kinases (BTK and JAK3) and molecules from the database. We positioned the docking box at the site of the eutectic small molecule, and the size of the two docking boxes was set to 20 × 20 × 20 Å. Low affinity indicates the likeliness of the molecule to bind better to the target protein. Therefore, the value of docking affinity can allow us to further eliminate inactive compounds. According to the docking binding energy, we individually ranked the compounds of BTK and JAK3 and removed those with positive and high affinities.

**Figure 5:**
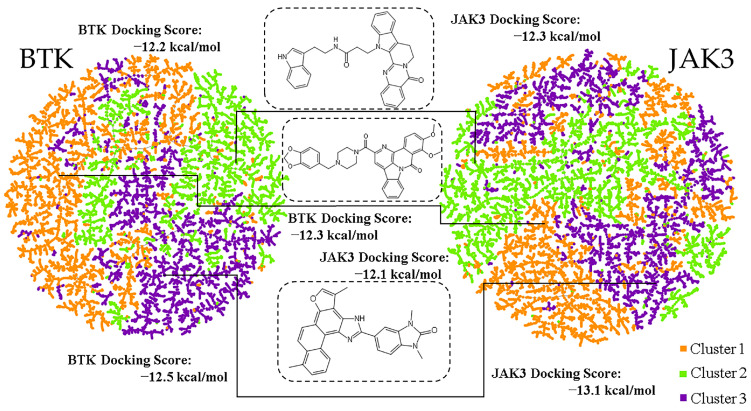
The three clusters are colored orange, green, and violet, respectively, for each target.

We speculated that selection of kinase inhibitor candidates from those with high ranks in binding energy might lead to redundant analogs. Therefore, we performed clustering [36] to obtain low-affinity representative molecules to increase molecular structural diversity. The downscaled molecular fingerprints were used as inputs for clustering analysis (Figure 5). To discover the dual-target inhibitors of BTK and JAK3, we identified molecules with low docking binding energy toward both BTK and JAK3. Finally, three natural product molecules from three different clusters and with low binding energy were selected.

### 2.5. ADMET Analysis

We subjected the screened compounds with low affinity toward the target proteins to ADMET analysis. Two free online tools, SwissADME (http://www.swissadme.ch/, accessed on 2 April 2023) and ADMETlab 2.0 (https://admetmesh.scbdd.com/, accessed on 2 April 2023), were used to determine the ADMET properties of the compounds, including the oil–water distribution coefficient (LogP), human intestinal absorption (HIA), skin penetration rate (LogKp), and Lipinski rule. In addition, solubility (LogS) and synthetic accessibility (SA) scores were calculated using RDKit (version 2022.09.1).

As shown in Table 4, all selected candidates followed the Lipinski rule and exhibited good synthesizability (SA score < 6; the smaller the value, the easier it is to synthesize compounds). In terms of the physicochemical properties of the screened molecules, the LogP value was in the range of 0.7–6.0, indicating their hydrophobic nature and the easy accessibility of the hydrophobic pocket of the proteins. The LogS value was around −6 (insoluble < −10 < not easily soluble < −6 < soluble), which indicated the solubility of the molecules in water. Considering the pharmacokinetic properties of HIA, all molecules showed a high probability of being absorbed in the intestine while not being easily permeable through the skin (the more negative the LogKp value, the lower the skin permeability). These prediction results indicate the acceptable ADMET properties of these selected potential inhibitors.

### 2.6. Molecular Dynamics Simulations

To explore the stability of the docking complex and its specific interaction, we conducted molecular dynamics simulations using Gromacs 2020. First, we performed molecular dynamics simulations of BTK and JAK3 with the inhibitors pirtobrutinib and peficitinib, respectively, for 100 ns, which served as a reference for subsequent complex system simulations. Next, using the docked conformation as the initial structure, we simulated the three selected molecules for 100 ns in BTK and JAK3 complexes. Finally, a total of eight sets of simulation results were used for subsequent analysis.

#### Root Mean Square Deviation (RMSD)

**Figure 6:**
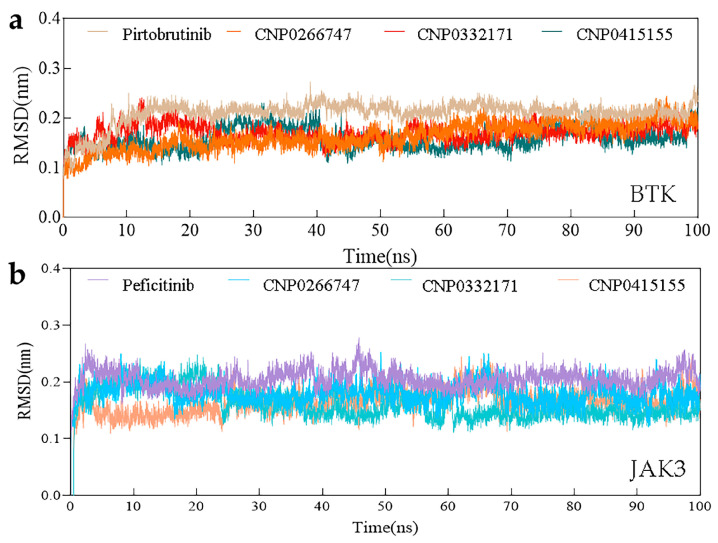
The changes in RMSD with time in the simulation. (**a**) RMSD values of BTK complex systems. (**b**) RMSD values of JAK3 complex systems.

RMSD measures the stability of a protein–ligand bond. In general, high values of RMSD are indicative of more dramatic alterations during the simulation process. We analyzed changes in complexes from the start conformation to the end location using RMSD. All complexes exhibited low RMSD values (less than 0.3 nm) throughout the process of simulation. We observed smooth RSMD curves over a long time period in the entire complex system (Figure 6), which implied an equilibrium. These eight RMSD curves remained largely consistent, except for some slight fluctuations and immediate rebalancing. In summary, overall RMSD analyses demonstrated that the complexes were at equilibrium after 10 ns of simulation.

### 2.7. MM/PBSA Binding Free Energy

We compared the binding abilities of the compounds and obtained the binding free energy values using the MM/PBSA method. As listed in Table 5, the binding free energy values of the two co-crystal complex systems BTK and JAK3 were −30.021 and −34.152 kcal/mol, respectively. The binding free energies of the three screened compounds toward their respective target protein were less than or close to the values of the binding free energies of the corresponding co-crystal complex systems. Thus, the screened compounds were all stabilized in the corresponding complex systems. The total binding free energy comprises van der Waals energy (E_vdw_), electrostatic energy (E_ele_), polar solvation (G_PB_), and nonpolar solvation (G_NP_). Herein, van der Waals energy was the largest component for the binding of compounds with BTK and JAK3.

**Figure 7:**
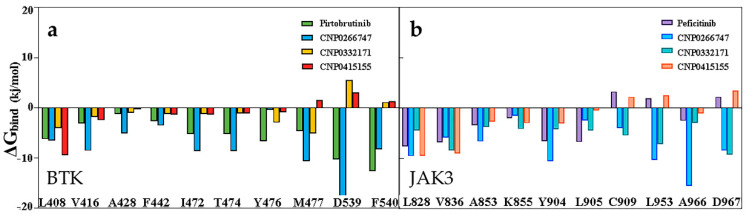
(**a**) Decomposition of the active site residues for binding free energy BTK complex systems. (**b**) Decomposition of the active site residues for binding free energy JAK3 complex systems.

We investigated the residue contribution of the binding free energies by performing binding free energy decomposition analysis and explored the interaction between the ligand and protein. For BTK, most residues except Met477, Asp539, and Phe540 showed a consistent trend in their contribution to the binding energy (Figure 7). The residue Met477 positively contributed to the CNP0266747, CNP0332171, and pirtobrutinib complexes. Only the residues Asp539 and Phe540 presented favorable contributions for binding to CNP0266747 and pirtobrutinib. For JAK3, we observed a consistent trend in the contribution of most residues to binding energy, with the exception of residues Cys909, Arg953, and Asp967 (Figure 7). As shown in Figure 7, residues Cys909 and Asp953 displayed positive contributions for binding to CNP0266747 and CNP0332171, and the residue Ala966 displayed a large energy gap in the contribution to the binding of CNP0266747. In conclusion, the binding free energy findings prove that all the compounds bound tightly to the target proteins in the simulation process. The analysis of residue contributions exposed the differences in contributions of key active site residues in the respective targets.

### 2.8. Interactions of the Screened Compounds with Their Protein Targets

**Figure 8:**
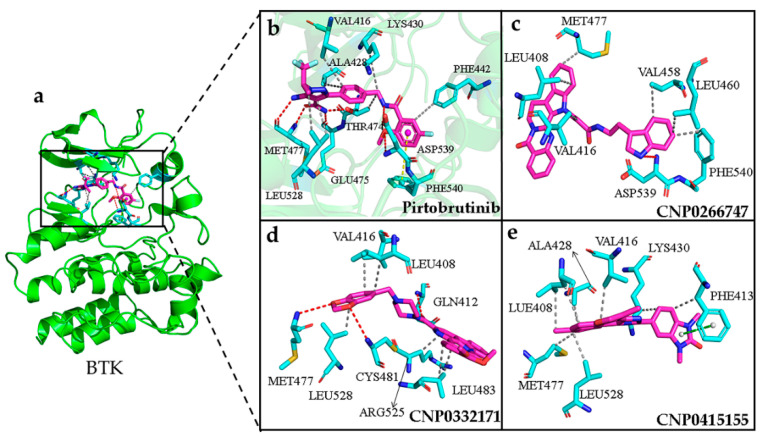
(**a**) The conformation of pirtobrutinib (shown in violet color and stick model) inside the active pocket of BTK (shown in green color and cartoon model). (**b**) Pirtobtutinib, (**c**) CNP0266747, (**d**) CNP0332171, (**e**) CNP0415155 inside in the active pocket of target protein. The compounds are shown as violet-colored sticks and the active residues are shown as cyan sticks. Hydrogen and hydrophobic bonds are formed between proteins and the compounds are shown as red and gray dotted lines, respectively. The pi–pi perpendicular and parallel stacking interactions are shown as yellow and green dotted lines, respectively.

**Figure A3:**
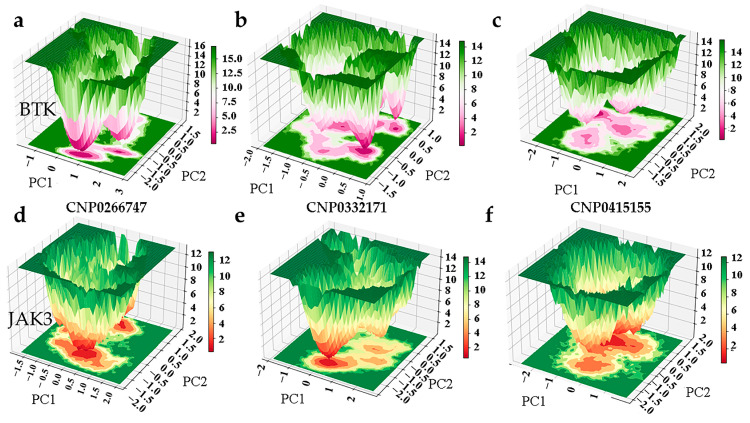
(**a**) The free energy landscape map of BTK-CNP0266747 complex system. (**b**) The free energy landscape map of BTK-CNP0332171 complex system. (**c**) The free energy landscape map of BTK-CNP0415155 complex system. (**d**) The free energy landscape map of JAK3-CNP0266747 complex system. (**e**) The free energy landscape map of JAK3-CNP0332171 complex system. (**f**) The free energy landscape map of JAK3-CNP0332171 complex system.

To study the reason underlying the differences in the binding affinities of compounds to their protein targets, we used the structure with the lowest free energy to analyze the interaction [37] (Figure A3). For BTK, the active sites included Val416, Ala428, Lys430, Phe442, Thr474, Glu475, Met477, Leu528, Asp539, and Phe540 residues, as detected from the interaction between BTK and pirtobrutinib. The key residues in the active sites included Met477, Lys430, Asp539, and Phe540 [38] (Figure 8a,b). The residue Met477 was located in the hinge region, while Asp539 and Phe540 were situated at the DFG motif. The hinge region forms important hydrogen bonds with ATP and ATP-competitive inhibitors, and the DFG motif domain comprises three conserved residues where D is involved in binding with activated-state Mg ions and F participates in the formation of activated-state R-spines. These findings further illustrate the importance of key amino acids in terms of structure.

Considering BTK, all interactions of the screened compound complexes at the active sites were consistent with those observed for the BTK–pirtobrutinib complex. H-bonds or hydrophobic interactions were found between the compounds and the hinge region, which provided them with stability in the active pocket (Figure 8a,b). The compound CNP0266747 formed one H-bond with Asp539 and six hydrophobic interactions with residues Leu408, Val416, Val458, Met477, Leu460, and Phe540 (Figure 8c). The residue Met477 located in the hinge region formed a hydrophobic interaction with the compound, while residues Asp539 and Phe540 from the DFG motif domain formed an H-bond and a hydrophobic interaction, respectively. MD simulation revealed the persistent presence of an H-bond with Asp539 (Table A1). The compound CNP0332171 formed three H-bonds with residues Gln412, Met477, and Cys481 and six hydrophobic interactions with residues Leu408, Val416, Leu483, Arg525, and Leu528 (Figure 8d). The compound CNP04151447 formed hydrophobic interactions with residues Leu408, Phe413, dal416, Ala428, Lys430, Met477, and Leu528. One pi–pi stacking interaction was formed between the benzene group of the compound and the residue Phe413 (Figure 8e). All these compounds can form sustained hydrogen bonds or hydrophobic interactions with the key residue Met477. Noteworthy, only the compound CNP0266747 formed interactions with the residues Asp539 and Phe540. This difference may be responsible for the different trends in the contribution of the residues to the binding free energy.

**Figure 9:**
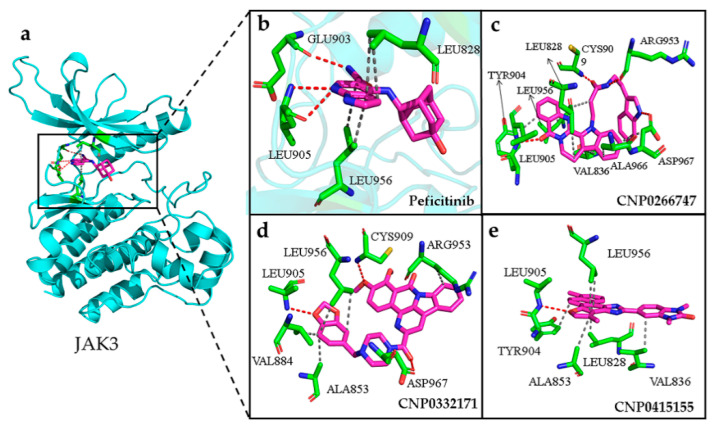
(**a**) The conformation of peficitinib (shown in violet color and stick model) inside the active pocket of JAK3 (shown in cyan color and cartoon model). (**b**) Peficitinib, (**c**) CNP0266747, (**d**) CNP0332171, (**e**) CNP0415155 inside the active site of target protein. The compounds are shown as violet-colored sticks and the active residues are shown as green sticks. Hydrogen and hydrophobic bonds are formed between proteins and the compounds are shown as red and gray dotted lines, respectively.

For the target JAK3, the active sites comprised the residues Leu828, Val836, Ala853, Lys855, Met902, Glu903, Leu905, Cys909, Arg953, Leu956, and Asp967 [39]. The binding sites on JAK3 for all compounds lay in the active sites. The JAK3–peficitinib complex mainly showed three persistent H-bonds with residues Glu903 and Leu905 from the hinge region that maintained stability throughout the simulation (Figure 9a,b). The importance of the formation of interactions between compounds and active sites from the hinge domain was demonstrated. As shown in Figure 9, all screened compounds formed stable hydrogen bonds with Glu903, Leu905, or Cys909 in the hinge region and stabilized their own structures in the binding pocket (Table A1). JAK3 is highly conserved in the binding pocket with other JAK family members, except at residues Cys909 and Ala966. Given the very highly conserved active pocket residues of the JAK family, the only differences included the residue Cys909 from the hinge region and Ala966 close to the DFG motif, which gathered our interest (Figure 9c–e). The compounds CNP0266747 and CNP0332171 could form H-bonds with Cys909 from the hinge region, Arg953 from the loop domain, and Asp967 from the DFG motif (Figure 9c,d). The compound CNP0266747 formed a hydrophobic interaction with the residue Ala966, which explains the difference in the contributions of the binding free energies of residues Cys909, Arg953, and Asp967 in residue decomposition and the large energy gap in the value of the residue Ala966 from the DFG motif region. This was the reason for differences in the contributions of residue free energy in MM/PBSA analysis.

The remaining BTK– and JAK3–compound interactions were all hydrophobic. Based on the analysis of the above interactions, in our MD results, the interactions formed by the compounds were mainly hydrophobic. This observation explains why the contribution of van der Waals energy was the largest among the components of the total binding free energy in all complex systems. In conclusion, the screened compounds could form H-bonds with the hinge region and stabilize their own structures in the binding pocket. Although the interacting residues were slightly different, they were located in the active pocket.

**Figure A4:**
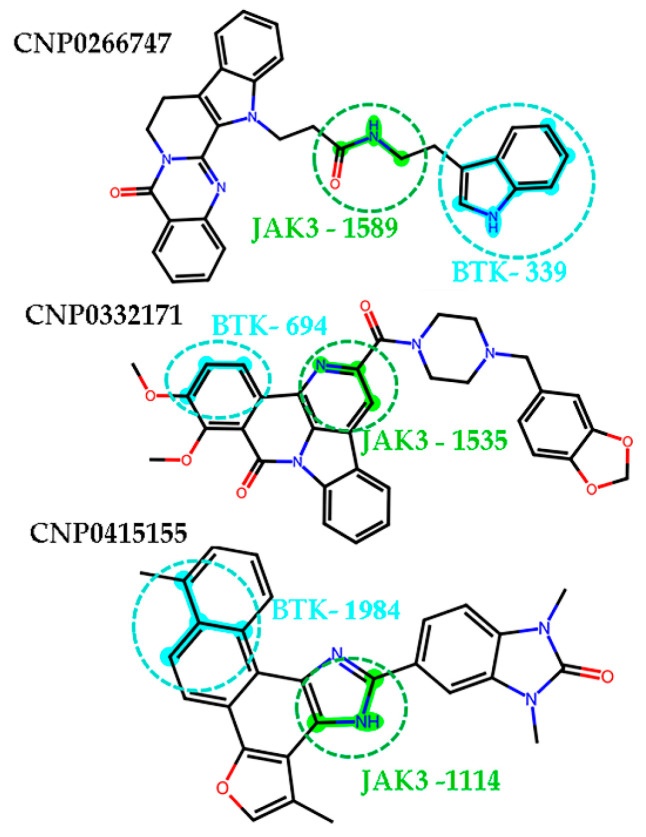
The active fingerprint fragments of the screen compounds are shown as cyan (BTK) highlights and green (JAK3) highlights.

We observed that the two-dimensional structure of the screened compounds comprised active fingerprint fragments of BTK and JAK3, which were mentioned in the context of the explanatory analysis of the models. The compound CNP0266747 included the 339 fingerprint fragment, displayed in cyan, and the 1589 fingerprint fragment, shown in green, against BTK and JAK3, respectively (Figure A4). It was interesting to observe that these fingerprint fragments could separately form both H-bonds and hydrophobic interactions with BTK and JAK3. Although CNP0332171 and CNP0415155 also contained fingerprint fragments 694 and 1984 for BTK and fingerprint fragments 1535 and 1114 for JAK3, respectively, they could only form hydrophobic interactions with BTK and JAK3. Only the active fingerprint fragments of CNP0266747 formed H-bonds and hydrophobic interactions with BTK and JAK3. Therefore, we hypothesized that this causes differences in the combining method with the protein–ligand complex. To sum up, each of the screened molecules contained active fingerprint fragments against both BTK and JAK3. This result further demonstrates that our models were reasonable and that the active fingerprint fragments we obtained via Shapley values were meaningful and accurate.

### 2.9. Active Fingerprint Fragments in CNP0266747 for Dual Targets

**Figure 10:**
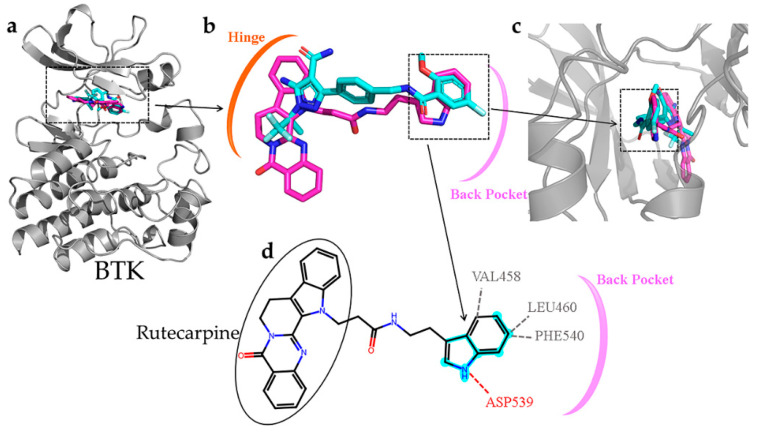
(**a**–**c**) The superimposition of the BTK-pirtobrutinib and BTK-CNP0226747 complexes. The protein is shown as gray cartoon. Cyan-colored sticks indicate pirtobrutinib and violet-colored sticks indicate compound CNP0226747. (**d**) Hydrogen and hydrophilic bonds that are formed between protein and the two-dimensional structure of the compound CNP0226747. Hydrogen and hydrophilic bonds are shown as red and gray dotted lines, respectively. The key molecular fingerprint fragment is shown as cyan highlight.

The compound CNP0266747 is a derivative of rutecarpine and exhibits anticancer and analgesic effects. Therefore, its binding to the two targets warrants further investigation. CNP0266747, with the lowest binding free energy against BTK and JAK3, had a special binding mode as compared with the other screened compounds (Figure 10a). Therefore, we thought it was worthwhile to explore the relationship of CNP0266747 with BTK and JAK3. For BTK, pirtobrutinib is a third-generation inhibitor that exhibits acceptable safety and selectivity profiles. The compound CNP0266747 had a consistent conformation in terms of spatial orientation with pirtobrutinib (Figure 10b,c). As shown in Figure 10c, we inserted the structure into the back pocket by bonding it with the surrounding residues. As we inserted the compound into the back pocket, its elution rate decreased and selectivity increased. Therefore, we suggest that the compound CNP0266747 showed selectivity for the BTK target, as observed with pirtobrutinib. Further analysis of the binding model of the compound CNP0266747 revealed that its head anchored to the molecule by forming a hydrophobic interaction with the hinge region residues Met477 and Leu408 (Figure 10d). The tail of CNP0266747 is an indole ring, which formed an H-bond with the residue Asp539 and hydrophobic interactions with residues Phe540 and Leu460. Interestingly, we found that the indole ring contained the fingerprint fragment 339 (cyan highlight) with top Shapley values. The fingerprint fragment 339 could bind to residues Asp539, Phe540, and Leu460, which facilitated the insertion of the molecular tail into the back pocket and consequently increased the selectivity of CNP0266747 (Figure 10d). Therefore, this fingerprint fragment has a decisive role for the BTK target in our opinion.

**Figure 11:**
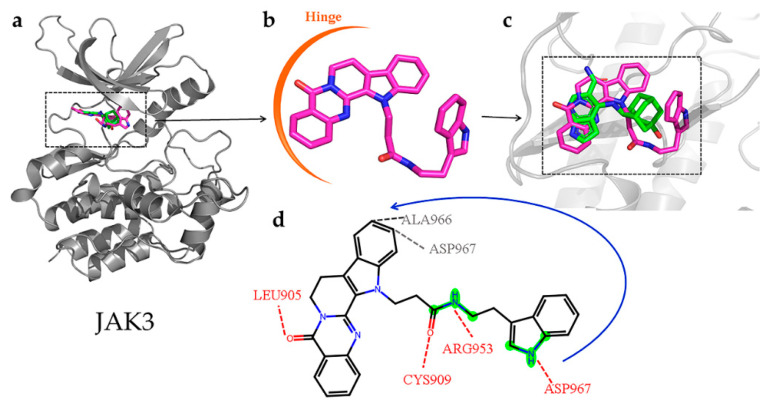
(**a**–**c**) The superimposition of the JAK3-peficitinib and JAK3-CNP0226747 complexes. The protein is shown as gray cartoon. Green-colored sticks represent pirtobrutinib and violet-colored sticks represent compound CNP0226747. (**d**) Hydrogen and hydrophilic bonds are formed between protein and the two-dimensional structure of the compound CNP0226747. Hydrogen and hydrophilic bonds are shown as red and gray dotted lines, respectively. The key molecular fingerprint fragment is shown as green highlight.

**Figure 12:**
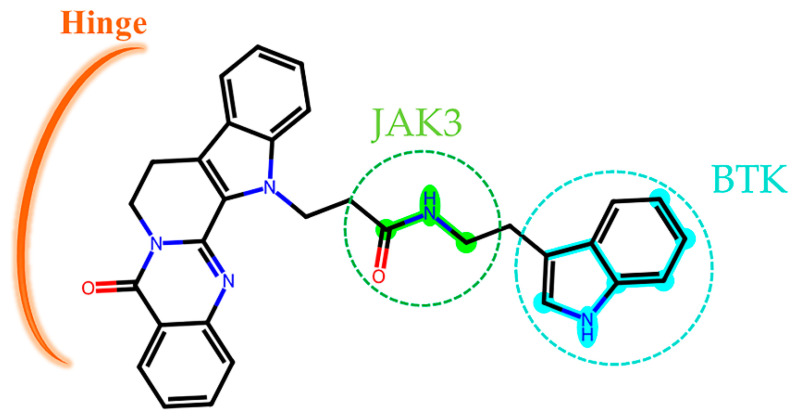
The two-dimensional structure of compound CNP0226747. The key molecular fingerprint fragments are shown as green and cyan highlights.

For JAK3, the rutecarpine fragment of CNP0266747 interacted with residues Leu828, Leu905 (the hinge region residue to stabilize the molecule), and Leu956 as observed with peficitinib, which was not a selective inhibitor (Figure 11a). As CNP0266747 exhibits a long aliphatic chain connecting to the indole ring, it may interact with additional residues in the active pocket, including Cys909, Arg953, Ala966, and Asp967. The residue Asp967 caused the compound CNP0266747 to form an O-shaped spatial conformation through hydrogen bonding and hydrophobic interaction with the head and tail of the compound (Figure 11b,c). This conformation allowed the compound to form a hydrophobic interaction with Ala966. Meanwhile, residues Cys909 and Arg953 further supported the O-conformation by forming hydrogen bonds with the long chain. As shown in Figure 11d, the fingerprint fragment 1589 (green highlight) with a high Shapley value played an important role in the formation of O-conformation, as it formed lasting H-bonds with Cys909, Arg953, and Ala966 and stabilized the special conformation. In summary, the compound CNP0266747 can form interactions with JAK3 in an O-conformation with the unique residues Cys909 and Ala966 via fingerprint fragmentation 1589. Although there was no co-crystallized structure for selective JAK inhibitors, CNP0266747 could interact with residues that were unique to JAK3. Therefore, we thought that CNP0266747 could possibly exhibit selectivity. The fingerprint fragmentation 1589 has an important role in the JAK3 structure. The compound CNP0266747 binds not only to BTK via fingerprint fragment 339 but also to JAK3 via fingerprint fragment 1589, and exhibits selectivity for both targets at the same time (Figure 12). In summary, the special binding mode of the compound CNP0266747 to BTK and JAK3 led to a free energy gap with other screened compounds, and its active fingerprint fragments 339 and 1589 played important roles in the formation of the special binding pattern.

## 3. Discussion

In the field of kinase drug discovery, researchers are actively seeking new methodologies to minimize wastage and reduce drug expenses. Multitargeted therapies have been the focus of research in this direction. However, this increases the complexity of this study. Herein, we introduce a general pipeline that integrates machine learning, the interpretable SHAP model, and molecular dynamics simulations to discover a dual-target drug candidate. BTK and JAK3 are two important enzymes that can be potentially targeted to inhibit downstream signaling pathways related to cancer cell growth. This study aimed to discover dual-target potential inhibitors of BTK and JAK3 from a natural product database. We established three machine learning models (RF, ET, and XGB) and validated their excellent activity prediction abilities. The three most important fingerprint features were listed as per the Shapley values. A 1:1:1 weighting strategy was used to classify the activity or inactivity of compounds from the natural product database using the three models. Three compounds (CNP0266747, CNP0332171, and CNP0415155) were selected as candidate BTK-JAK3 dual-target inhibitors via molecular docking, clustering, and ADMET analyses. Finally, we performed molecular dynamics simulations and MM/PBSA to calculate their binding free energies. These compounds could stably bind to both targets by forming H-bonds and hydrophobic interactions. All of the screened compounds contained active fingerprint fragments against BTK and JAK3. The compound CNP0266747 was chosen as the focus of this work, as it exhibited potent binding free energy and different residue combinations against BTK and JAK3. For BTK, Met477, Asp539, and Phe540 were key amino acids that facilitated the insertion of the compound into the back pocket. For JAK3, the residues Cys909, Arg953, Ala966, and Asp967 were involved in the stabilization of the O-conformation of the protein−inhibitor complex. CNP0266747 has unique binding modes for two targets. Moreover, its fingerprint features 339 and 1589 played crucial roles during the process of binding with the key residues. In conclusion, this work is an attempt to develop a general pipeline that predicts the candidate dual inhibitors of BTK and JAK3 and provides helpful guidance for drug design. CNP0266747 displays huge potential as a dual-target inhibitor of BTK and JAK3 and is expected to undergo follow-up research.

## 4. Materials and Methods

### 4.1. Collection and Preparation of Data

We collected the IC_50_ values of active compounds against BTK and JAK3 from the BindingDB database (https://bindingdb.org/bind/index.jsp, accessed on 5 October 2022). Duplicates and inactive compounds were excluded to obtain 15,438 BTK-active compounds and 8846 JAK3-active compounds. As activating molecules, compounds with IC_50_ values < 100 nM were flagged as active molecules and those with IC_50_ values > 100 nM were flagged as inactive molecules. Data were relatively balanced and then standardized with RDKit. The Coconut natural product database (https://coconut.naturalproducts.net/, accessed on 10 January 2023) was used for virtual screening of the compounds.

### 4.2. Model Construction

The molecular descriptor is the result of a process that converts chemical information into mathematical numbers. We used RDKit to calculate ECFPs [40], which are represented by a set of integer identifiers of indefinite length and act as the most primitive and accurate representation. Each identifier represents a specific substructure. ECFPs extract the features of the current layer by stitching the features in the neighborhood of the previous layer and then using a fixed hash function. The result is considered as an integer index, and then the vertex feature vector is filled in at the position corresponding to index 1. In total, 2048 molecular fingerprints were computed to construct machine learning models.

After preliminary exploration, the IC_50_ value distribution was discrete. Thus, we converted IC_50_ values to pIC_50_ values. After this transformation, the data were split into training and test sets at a 4:1 ratio. The same random seed was used on the data to assure consistent data segmentation. Random forests (RFs) [41], extra trees (ETs) [42], and XGBoost (XGB) [43] were selected for model construction because of their high effectiveness and robustness. Finally, Bayesian optimization [44] and tenfold cross-validation were employed to determine the best parametric model.

### 4.3. Evaluation and Explanation of Models

**Figure A5:**
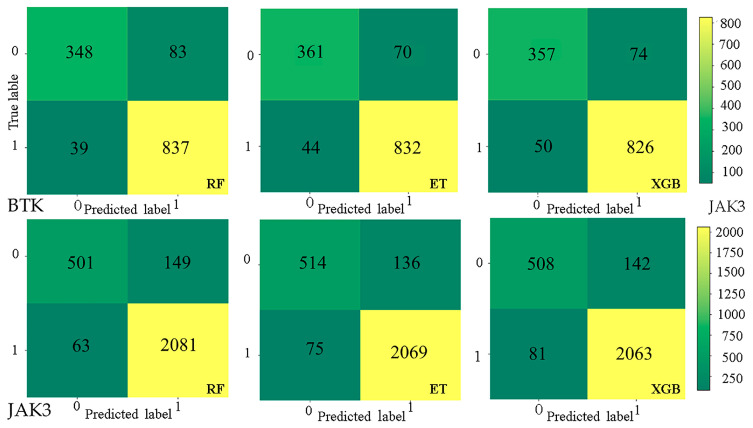
Confusion matrix for the test sets of RF, ET, and XGB models against BTK and JAK3, respectively.

We selected five parameters to evaluate the performance of the models, namely area under the curve (AUC), accuracy (ACC), precision (Pre), F1 score (F1), and recall. All of these parameters except AUC can be derived from a confusion matrix, which is the summary of prediction results for classification problems that uses a counting method to enumerate the correct and incorrect quantities (Table 6). It is divided into true positive (TP), false positive (FP), false negative (FN), and true negative (TN) predictions (Figure A5).

The five parameters, except AUC, can be obtained from the following equations:

(1) $$Pre=\frac{TP}{TP+FP}$$

(2) $$Se=\frac{TP}{TP+FN}$$

(3) $$Recall=\frac{TP}{TP+FN}$$

(4) $$F1=\frac{2\times Pre\times Se}{Pre+Se}$$

(5) $$Acc=\frac{TP+TN}{TP+FP+TN+FN}$$

Pre indicates the percentage of actual prediction of positive samples in the prediction of positive samples, Se indicates the percentage of actual prediction of positive samples in the actual positive samples, Recall reflects the percentage of prediction of positive samples in the actual positive samples, F1 reflects the relationship between precision and recall, Acc reflects the degree of model accuracy, and the AUC of the ROC curve is an important indicator of a good or bad model. All parameters were as close to 1 as possible.

### 4.4. Model Interpretation

The interpretability of models plays an important role in practical applications owing to the black-box effect generated by machine learning, which may limit the applications of computerized decisions [45]. SHAP is an approach derived from “Cooperative Game Theory” employed to address model interpretability. It explains the importance of sample characteristics where compensation is related to respective contribution [46]. This method is important in interpreting the ranks of the model features at the end. It is used to interpret the importance of sample characteristics to the model and the influence of features on the model’s decision directions. Considering these advantages, we adopted SHAP as the method to explain our models.

### 4.5. Virtual Screening

AutoDock Vina was employed for executive molecular docking of BTK (PDB ID: 8FLL) and JAK3 (PDB ID: 6AAK) with the Coconut database filtered via machine learning models, respectively [47]. The docking box center was located at the small molecule of the inhibitor in the crystal structure, and the box size was set to 20 Å × 20 Å × 20 Å. Before docking, Jackal software was used to complete the missing protein residues and atoms and to add polar hydrogen for protein pretreatment. After docking, the compounds were clustered into three categories using the Kmeans classification method [48].

ADMET is very important in contemporary drug design and screening. ADMET prediction serves as a basic criterion to assess the nature of druglike substances. We predicted ADMET properties with the help of SwissADME [49] (http://www.swissadme.ch/, accessed on 2 April 2023) and ADMETlab 2.0 [50] (https://admetmesh.scbdd.com/http://www.swissadme.ch/, accessed on 2 April 2023).

### 4.6. Molecular Dynamics Simulations

We used the Gromacs 2020 package for molecular dynamics simulations. We used Gaussian16 software to calculate the compounds’ RESP (restrained electrostatic potential) charges [51]. Then, we generated the parameter files for the Amber force field using AmberTools21 [52]. Finally, RESP charges [53] were used to replace the original charge in the generated file.

We constructed a protein–ligand complex system with the screened small molecules and proteins using Amber force field, TIP3P water model, and added Na^+^ and Cl^+^ as counteracting ions [54]. The steepest descent method was employed to optimize energy to obtain the lowest energy conformation. An isovolumetric–isothermal NVT equilibrium of 200 ps was performed at 310 K, and an isothermal–isobaric NPT equilibrium of 200 ps at 1 atm was performed. Finally, molecular dynamics simulations were performed at 100 ns. The LINS algorithm was employed to constrain the bond during the process of simulation, and the particle mesh Ewald (PME) method [55] was applied to the long-range electrostatic field.

After the simulation, the gmx_MMPBSA tool was used to calculate the binding free energy (∆G_bind_) [56]. It was composed of three energetic terms, namely potential energy in vacuum (ΔE_MM_), polar solvation energy (ΔG_GB_), nonpolar solvation energy (ΔG_SA_), and TΔS (the entropy contribution at temperature T) [57,58]. The energy was calculated using the following equation:

(6) $$\begin{array}{cc} {∆G}_{bind} & =∆E_{MM}+∆G_{GB}+∆G_{SA}-T∆S\\ & =∆E_{vdw}+∆E_{ele}+∆G_{GB}+∆G_{SA}-T∆S \end{array}$$

## Figures and Tables

**Table 1:** Statistical results of BTK and JAK3 classification models on training sets (tenfold cross-validation).

	Method	AUC	Pre	F1	Recall	ACC
BTK	RF	0.9487	0.9287	0.9491	0.9705	0.9195
	ET	0.9355	0.9327	0.9451	0.9579	0.9139
	XGB	0.9524	0.9340	0.9483	0.9631	0.9187
JAK3	RF	0.9570	0.9111	0.9321	0.9543	0.9043
	ET	0.9400	0.9196	0.9313	0.9435	0.9044
	XGB	0.9650	0.9209	0.9358	0.9513	0.9102

**Table 2:** Statistical results of BTK and JAK3 classification models on test sets.

	Method	AUC	Pre	F1	Recall	ACC
BTK	RF	0.9605	0.9311	0.9536	0.9771	0.9270
	ET	0.9596	0.9346	0.9500	0.9700	0.9220
	XGB	0.9668	0.9406	0.9540	0.9678	0.9284
JAK3	RF	0.9640	0.9043	0.9313	0.9600	0.9051
	ET	0.9557	0.9184	0.9344	0.9509	0.9105
	XGB	0.9686	0.9181	0.9326	0.9475	0.9082

**Table 3:** Molecular fingerprint fragments. The symbol * represents that other groups can be attached here.

	Bit	Fragment	Center	Radius
BTK	339	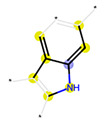	C	2
	694	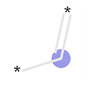	C	0
	1984	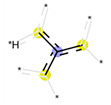	C	1
JAK3	1589	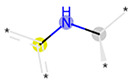	N	1
	1535	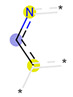	C	1
	1114	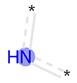	N	0

**Table 4:** The drug properties of screened compounds obtained with SwissADME, ADMETlab2.0, and RDkit.

Natural Compound	Physicochemical Properties		Pharmacokinetics		Druglikeness	SA
	LogP	LogS	HIA	logKp	Lipinski Rule	
CNP0266747	3.43	−5.70	98.0%	−6.35 cm/s	Accepted	3.96
CNP0332171	3.38	−6.24	99.8%	−6.69 cm/s	Accepted	3.93
CNP0415155	4.15	−6.82	86.4%	−4.91 cm/s	Accepted	3.51

**Table 5:** The binding free energies (kcal/mol).

	Compound	ΔE_ele_	ΔE_vdw_	ΔG_PB_	ΔG_NP_	−TΔS	ΔG_bind_
BTK	CNP0266747	−12.404	−57.269	32.323	−6.575	3.476	−40.236
	CNP0332171	−6.129	−57.993	34.256	−7.908	4.302	−32.754
	CNP0415155	−3.722	−54.622	30.340	−6.607	3.368	−31.245
	Pirtobrutinib	−20.240	−64.578	53.830	−7.248	4.084	−34.152
JAK3	CNP0266747	−12.972	−64.284	41.625	−7.083	5.248	−37.468
	CNP0332171	−10.516	−64.387	48.254	−7.075	3.445	−30.280
	CNP0415155	−1.905	−49.585	23.801	−6.073	2.483	−31.280
	Peficitinib	−5.918	−45.560	24.507	−5.379	2.362	−30.021

**Table 6:** Confusion matrix.

	Positive Prediction	Negative Prediction
**True positive**	True positive (TP)	False negative (FN)
**True negative**	False positive (FP)	True negative (TN)

## Data Availability

The data presented in this study are available in the Supplementary Materials.

## Supplementary Materials

The following supporting information can be downloaded at: https://www.mdpi.com/article/10.3390/molecules28207140/s1.

## Appendix A

**Table A1:** The key hydrogen bond occupancies at the protein–ligand binding sites.

	Compound	Residues	Occupancy		Compound	Residues	Occupancy
BTK	Pirtobrutinib	Glu475 Met477 Asp539	99.3% 95.7% 41.1%	JAK3	Peficitinib	Glu903 Leu905	94.5% 77.8%
	CNP0266747	Asp539	97.6%		CNP0266747	Asp967 Leu905 Cys909 Arg953	90.2% 97.9% 50.2% 42.9%
	CNP0332171	Gln412 Met477	55.2% 75.3%		CNP0332171	Leu905 Cys909	88.3% 20.4%
	CNP0415155	Lys430	12.3%		CNP0415155	Leu905	58.5%
